# Surgical face masks do not impair the decoding of facial expressions of negative affect more severely in older than in younger adults

**DOI:** 10.1186/s41235-022-00403-8

**Published:** 2022-07-16

**Authors:** Lea Henke, Maja Guseva, Katja Wagemans, Doris Pischedda, John-Dylan Haynes, Georg Jahn, Silke Anders

**Affiliations:** 1 Department of Neurology, Universität zu Lübeck, Ratzeburger Allee 160, Lübeck, Germany; 2 Center of Brain, Behavior and Metabolism (CBBM), Universität zu Lübeck, Lübeck, Germany; 3 Department of Psychology, Universität zu Lübeck, Lübeck, Germany; 4 Bernstein Center for Computational Neuroscience, Charité – Universitätsmedizin Berlin, Corporate Member of Freie Universität Berlin and Humboldt-Universität zu Berlin, Berlin, Germany; 5 Science of Intelligence, Research Cluster of Excellence, Technische Universität Berlin, Berlin, Germany; 6 Berlin School of Mind and Brain, Humboldt-Universität zu Berlin, Berlin, Germany; 7 Department of Psychology, Humboldt-Universität zu Berlin, Berlin, Germany; 8 Department of Psychology, Chemnitz University of Technology, Chemnitz, Germany

**Keywords:** Online study, Facial emotion recognition, Face mask, Anger, Fear, Contempt, Sadness, Age, COVID-19, Pandemic

## Abstract

Surgical face masks reduce the spread of airborne pathogens but also disturb the flow of information between individuals. The risk of getting seriously ill after infection with SARS-COV-2 during the present COVID-19 pandemic amplifies with age, suggesting that face masks should be worn especially during face-to-face contact with and between older people. However, the ability to accurately perceive and understand communication signals decreases with age, and it is currently unknown whether face masks impair facial communication more severely in older people. We compared the impact of surgical face masks on dynamic facial emotion recognition in younger (18–30 years) and older (65–85 years) adults (N = 96) in an online study. Participants watched short video clips of young women who facially expressed anger, fear, contempt or sadness. Faces of half of the women were covered by a digitally added surgical face mask. As expected, emotion recognition accuracy declined with age, and face masks reduced emotion recognition accuracy in both younger and older participants. Unexpectedly, the effect of face masks did not differ between age groups. Further analyses showed that masks also reduced the participants’ overall confidence in their emotion judgements, but not their performance awareness (the difference between their confidence ratings for correct and incorrect responses). Again, there were no mask-by-age interactions. Finally, data obtained with a newly developed questionnaire (*attitudes towards face masks, atom*) suggest that younger and older people do not differ in how much they feel impaired in their understanding of other people’s emotions by face masks or how useful they find face masks in confining the COVID-19 pandemic. In sum, these findings do not provide evidence that the impact of face masks on the decoding of facial signals is disproportionally larger in older people.

**Supplementary Information:**

The online version contains supplementary material available at 10.1186/s41235-022-00403-8.

## Significance statement

Surgical face masks reduce the spread of airborne pathogens but also impair communication. The risk of getting seriously ill after infection with SARS-COV-2 during the present COVID-19 pandemic amplifies with age, suggesting that face masks should be worn especially during face-to-face contact with and between older people. However, facial signals help people to understand other people, and the ability to accurately perceive and understand facial expressions declines with age. In this online study we examined how face masks impair the understanding of facial signals of affect in older people. In sum, we found no evidence that face masks impair facial understanding disproportionally more strongly in older than in younger adults, neither at the behavioural level (as assessed with a facial emotion recognition task) nor at the subjective level (as assessed with a newly developed online questionnaire).

## Introduction

Face masks are physical barriers that cover a substantial part of the face (Carbon, 2020). While surgical face masks effectively reduce the transmission of airborne pathogens between individuals and thereby significantly lower the individual risk of getting infected with SARS-COV-2 during the current COVID-19 pandemic (Catching et al., 2021; Sommerstein et al., 2020), they also reduce the flow of information between individuals (Carbon, 2020; Bani et al., 2021; Cohn et al., 2021; Carbon & Sorreno, 2021; Gori et al., 2021; Grundmann et al., 2021; Kastendieck et al., 2022; Nicksic et al., 2021; Noyes et al., 2021). The threat of getting seriously ill after infection with SARS-COV-2 increases with age (Verity et al., 2020), suggesting that face masks should be worn especially during face-to-face contact with and between older people. However, faces are an important source of information during human interaction (Grahe & Bernieri, 1999; Jacob et al., 2013; Noller, 1985), and the ability to accurately perceive and understand facial signals declines with age (Ruffman et al., 2008; Henry et al., 2013; Goncalves et al., 2018; Hayes et al., 2020). To estimate the psychological costs of face masks in the older population studies are needed that quantify the effects of face masks on social perception in older people (Schroeter et al., 2021).

For facial expressions of emotions the negative correlation between age and decoding accuracy across the life span has been estimated to be as high as |*r*|= 0.46 (Schlegel et al., 2014). Furthermore, older people seem to depend more on information in the lower part of the face than younger adults when decoding facial expressions. Older participants spend more time scanning the mouth region and less time scanning the eye region of facial expressions than younger participants (Grainger & Henry, 2020) and meta-analyses (Ruffman et al., 2008; Goncalves et al., 2018; Hayes et al., 2020) consistently show that older participants experience less difficulties when facing expressions that carry almost all relevant information in the mouth region (i.e., happiness, disgust; Smith et al., 2005; Wegrzyn et al., 2017) than when facing expressions that carry important information in the eye region (i.e., anger, fear; Smith et al., 2005; Wegrzyn et al., 2017). In line with this, there is preliminary evidence that face masks impair facial emotion recognition more severely in older than in younger adults (Grundmann et al., 2021).

The current study was designed to examine and compare the effect of surgical face masks on facial emotion recognition accuracy and confidence in a younger (18–30 years) and an older (65–85 years) sample that were well-balanced with respect to sociodemographic factors (gender, education, occupation). Participants judged videotaped facial expressions (anger, fear, sadness and contempt) of twelve female models whose face was either fully visible or partly covered by a digitally added surgical face mask in a forced choice design. After each judgement, participants were asked to rate how confident they were that their response was correct. In addition to emotion recognition accuracy and confidence, we assessed the participants’ performance awareness. Performance awareness was estimated as a participant’s confidence ratings in trials in which their emotion judgement was actually correct subtracted with their confidence ratings in trials in which their judgement was actually incorrect.

Because there is evidence that beliefs and attitudes can have an effect on emotion recognition accuracy (e.g., teachers who believe that anger is a harmful emotion in school contexts have been found to detect anger less accurately in children’s facial expressions than teachers who believe that anger is a useful emotion in school contexts; Hagan et al., 2020) and beliefs about the harms and benefits of face masks might vary across younger and older adults, we also assessed and compared younger and older adults’ attitudes towards face masks. For this, we used a newly developed questionnaire, the *atom* (*attitudes towards face mask*) questionnaire.

## Methods

### Participants

The study was conducted in Germany in spring/summer 2021, approximately one year after the German government issued the first face mask decree. Snowball sampling was used to recruit participants of different ages. Initially, eligible individuals in two co-authors’ (LH and SA) social networks were personally invited to participate. Individuals who completed the experiment were then asked to recruit further individuals from their own social network. Predefined inclusion criteria were age (between 18 and 30 or between 65 and 85 years old) and access to the internet via a personal computer or laptop. Predefined exclusion criteria were pre-existing psychiatric or neurological conditions, severe hearing loss, current medication with psychotropic drugs and non-German first languages. Biographical data of all individuals who had completed the experiment were monitored once per week to detect data sets that had to be excluded and to focus the recruitment process on individuals needed to balance age cohorts with respect to gender, education and occupation. One-hundred-and-eight individuals completed the experiment between April 24 and July 18, 2021. Three of these individuals did not comply with the age criterion and eight other individuals did not comply with the exclusion criteria; data from these individuals were excluded. In order to obtain equally-sized age cohorts, data of the last individual in the old cohort were also excluded. The final sample consisted of data from 96 participants, 48 per age cohort. Gender (24 women and 24 men per sample), education level (basic education [nine/ten years] / high school / university degree; young cohort, 4/30/14, old cohort, 5/32/11) and occupation (social/technical/administrative; young cohort 11/8/29, old cohort 10/12/26) were well matched between cohorts. Forty participants in the old cohort and no participant in the young cohort were retired at the time of participation.

### Facial stimuli

Video clips of facial expressions recorded and evaluated in a previous study (Broer, 2020) were used as stimuli. In short, university students (age 20–30 years) were invited to serve as models for a set of emotional facial expressions to be used in the future studies. In each trial, they were asked to recall and submerge themselves into an emotional situation they had experienced in real life, and to facially express their emotional feelings as they arose. Models were explicitly instructed not to pose emotions but to express their genuine affective feelings. Video recordings were obtained with a standard video camera (Sony HDR-CX560VE), positioned 1 m in front of the model’s face and self-operated by the model by mouse click. The start and end of each recording (20 s) was indicated by an acoustic signal. Six video recordings of four different emotions (*Wut* [anger], *Angst* [fear], *Traurigkeit* [sadness] and *Verachtung* [contempt]) were obtained from each model. Recordings of a given emotion were obtained in a row.

Six-hundred-twenty-eight video recordings from 28 female models were screened with regard to visual quality and intensity of the facial expression by four raters (students and researchers of the lab). For each model and emotion, three high-quality recordings were selected whose expressive intensity was rated between 10 and 60% by at least three of the four raters. The selected 336 video recordings were cut into clips of 8 s. An evaluation study with 84 naïve raters (age 19 to 24 years, 52 women, 32 men) was conducted to obtain emotion recognition accuracies for each clip (four-alternative forced choice, 26–30 raters per clip). Video clips of four models were very dissimilar with regard to mean emotion recognition accuracy (range from below 0.25 to above 0.95); video clips of these models were excluded. For each of the remaining 24 models the two video clips with the most similar mean recognition accuracies were selected for each emotion; all other video clips were discarded. In order to obtain stimulus sets with moderate variance, no video clips with mean accuracies below 0.25 [chance level] or above 0.80 were selected. The resulting 192 video clips (2 video clips per emotion × 4 emotions × 24 models) constitute the *LUV* (*LUebeck Video clips of affective facial expressions*).

Note that the LUV differs from other sets of facial expressions in some aspects. First, the LUV does not comprise facial expressions of happiness because facial expressions of happiness are usually very easily detected among negative emotions and might lead to imbalanced hit rates across emotions. Second, for the same reason, the LUV does not contain facial expressions of disgust but instead includes facial expressions of contempt. Facial expressions of contempt and disgust appear very similar (Thompson & Meltzer, 1964) and were originally considered a variant of the same basic emotion (Ekman et al., 1969). Third, in its current version the LUV consists of facial expressions of young adult women because facial expressions that carry an equal amount of affective information are difficult to obtain from men and older models. In the context of the current study it is important to note that there is currently no evidence that participants are better at decoding affective facial expressions of models of their own age (Sze et al., 2012).

**Fig. 1:**
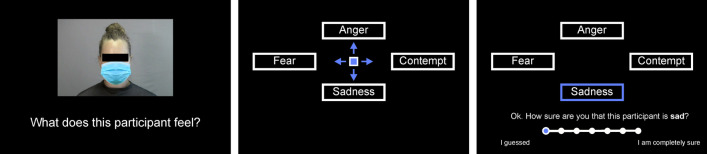
Time line of an emotion recognition trial. Participants saw video clips (8 s) of 12 different young female models expressing fear, anger, contempt or sadness. For each participant the lower part of the face of half of the models was covered with a digitally added surgical face mask (left). After each video a response screen appeared, asking participants to select the emotion they thought the person in the video had experienced (middle) by button press. After they had entered their response, participants were asked to rate how sure they were that their judgement was correct (right). In the figure, original German terms and texts are translated to English. Note that the black bar covering the model’s eye region was not shown in the video

For the current study, video clips of half of the models of the LUV were used to create two balanced subsets (*V* and *W*). For this, the 12 models were pairwise matched with regard to their emotion accuracy profile (the mean emotion recognition accuracy for each emotion in the evaluation study). All video clips of one model of a matched pair were assigned to one subset and all video clips of the other model to the other subset. Two versions of each subset were created, one version in which all video clips were left as they were and one version in which in each video clip the model’s face was partly covered by a digitally added surgical face mask (Fig. 1). Face masks were added frame-by-frame relative to a reference point on the model’s face so that they moved with the face (using *Adobe*, Adobe Inc.). Set *VW* contained unprocessed video clips of the six models of subset *V* and masked video clips of the six models of subset *W*, and set *WV* contained unprocessed video clips of the six models of subset *W* and masked video clips of the six models of subset *V*.

Two pseudo-randomized orders of the 96 video clips were created with the restriction that one video clip of each emotion and model was shown in trial 1–48, and the other one in trial 49–96. To avoid that participants learned the structure of the stimulus set, we additionally swapped *fear* clips of two models in trial 1–48 with *sadness* clips from trial 49–96, *sadness* clips of two models in trial 1–48 with *fear* clips from trial 49–96, *anger* clips of two models in trial 1–48 with *contempt* clips from trial 49–96, and *contempt* clips of two models in trial 1–48 with *anger* clips from trial 49–96. Video clips of each set (*VW* and *WV*) were shown in four different orders (the two pseudo-randomized orders were used forward and backward), resulting in a total of eight different stimulus versions (2 sets × 4 orders per set). Each participant saw one version, and versions were balanced over age cohorts and gender.

### Emotion recognition task

Each participant completed 96 emotion recognition trials (each video clip was used once). Each trial started with a cross hair (500 ms) followed by the video clip (8 s). Immediately after the video clip a response selection screen appeared with the four response options arranged clockwise around the cursor (*Angst* [fear, left], *Wut* [anger, top], *Verachtung* [contempt, right], *Traurigkeit* [sadness, bottom], Fig. 1). Participants selected their response by pressing the corresponding arrow head on the keyboard. The selected emotion was highlighted and a question appeared at the bottom of the screen asking the participant to rate how confident they were that the model expressed the selected emotion on a 7-point scale displayed below the question. Ratings were made by moving the cursor from the very left (anchor “I guessed”) to the right (anchor “I am completely sure”) with keys *P* (left) and *Q* (right) (Fig. 1). The next trial started as soon as the participant pressed Enter. There was no time limit but participants were instructed to respond quickly and intuitively.

Before and after the emotion recognition task, participants were asked to evaluate each model in random order with regard to (i) how trustable they thought the person was, (ii) how likable they found the person, (iii) how much they would like to meet the person in real life and (iv) how close they felt to the person. For models shown with masks only, participants were additionally asked to judge (v) how much they would like to see the person without a mask. Questions appeared one by one below a still picture of the corresponding model (with a neutral facial expression). Judgements were made on a 13-point visual scale displayed below each question by moving the cursor from the centre of the scale to the left (anchor “not at all”) or to the right (anchor “very much”) with keys *P* (left) and *Q* (right). Models were shown without or with the digitally added face mask as in the emotion recognition task.

The emotion recognition and interpersonal appraisal tasks were each introduced by a short written text that informed participants that they would see video recordings/pictures of participants of a previous study, followed by an online step-by-step instruction and two practise trials with models not included in the main part of the experiment. Emotion recognition and interpersonal appraisal trials were implemented on *JATOS* (Lange et al., 2015). *Want-to-meet* and *want-to-see-without-mask* data and all post-emotion recognition appraisal data were obtained as part of a different study and will not be reported here.

### Questionnaires

After the emotion recognition and interpersonal appraisal tasks participants were asked to complete two questionnaires. The first questionnaire, the *atom* (*attitudes towards face masks*), was specifically developed for the current study and designed to assess (i) how much people feel that face masks impair their understanding of others’ emotions (*perceived impairment*) and (ii) how much they believe face masks help to confine the pandemic (*perceived utility*). A pool of items was created for each of the two subscales following the rationale that attitudes can be measured at three levels (affect, behaviour, cognition; Breckler, 1984). Items were iteratively adapted with regard to their construct validity and intelligibility by two co-authors (LH and SA). The final *atom* questionnaire contained 12 items (Table S1 in Additional file 1 in the supplemental material). The second questionnaire comprised the 12 items of the *Social Curiosity Scale* (Renner, 2006) and the four extraversion and neuroticism items of the German version of the *Big Five Inventory* (Rammstedt & John, 2007). This questionnaire was used as part of a different study and scores will not be reported here. Questionnaires were implemented on *SoSci Survey* (https://www.soscisurvey.de).

### Procedure

All parts of the experiment were completed online. Interested individuals were contacted by phone or e-mail. Participants were offered that one of the co-authors (LH) would call them and stay on the phone while they completed the experiment to have technical support available when needed. If requested, the experimenter called the participant at an appointed time and stayed on the phone as long as desired. The emotion judgement / interpersonal appraisal part and the questionnaire part of the experiment could be completed in a row or with a break, but the emotion judgement / interpersonal appraisal part always had to be completed first. Biographical data (gender, year of birth, first language, education, occupation, whether or not they were retired, whether or not they had a history of psychiatric or neurological illness, whether or not they were currently taking psychotropic drugs, hearing ability) and consent were obtained before the emotion judgement / interpersonal appraisal part. Completion of the emotion judgement / interpersonal appraisal part took on average 50 min and completion of the questionnaire part took on average 15 min.

### Data analysis

Three emotion recognition measures were assessed: *accuracy, confidence* and *performance awareness*. Performance awareness was estimated as a participant’s confidence ratings in trials in which they had selected the correct response subtracted with their confidence ratings in trials in which they had selected an incorrect response (i.e. performance awareness = confidence _correct responses_ − confidence _incorrect response_). Two additional measures were computed to compare performance across emotions: *response frequency* and *unbiased hit rates*. A participant’s response frequency for a given emotion is the percentage of trials the participant selected that emotion, irrespective of whether the response was correct or incorrect. A participant’s unbiased hit rate *hu* for a given emotion is the participant’s hit rate for that emotion multiplied by the participant’s correct response rate for that emotion (i.e. *hu *_emo_ = [# correct responses _emo_ / # stimuli _emo_] × [# correct responses _emo_ / # responses _emo_]) (Wagner, 1993). Note that the chance level for an unbiased hit rate depends on the participant’s response frequency and is given by the overall chance level multiplied by the participant’s response frequency for that emotion (i.e. *hu *_chance emo_ = [1/ # of response options] × [# responses _emo_ / # trials]). ∆ hit rates in Table 1 and unbiased hit rates in Table 3 and Fig. 3 are subtracted with chance level, and confidence ratings are rescaled to unity.

To account for the fact that baseline performance (emotion recognition in facial expressions not covered by a face mask) might differ between younger and older participants we also attempted to estimate the *relative decline* in emotion recognition for each cohort and emotion. Because hit rates and unbiased hit rates can only be interpreted relative to chance level, we estimated the relative decline in hit rates by scaling the absolute decrease in hit rates / unbiased hit rates to baseline performance subtracted with chance (i.e. relative decline = hit rate _w/o mask–with mask_ / [hit rate _w/o mask_ − chance level] and relative decline = *hu*_w/o mask–with mask_ / [*hu*_w/o mask_ − response frequency x chance level]). However, because baseline accuracy was not greater than chance level in each and every participant these analyses could only be computed at group level and no statistical inferences could be drawn.

Bayes Statistics were computed for all other analyses. We predicted that emotion recognition (accuracy, confidence, performance accuracy) (i) would decline with age, (ii) would be impaired by face masks and (iii) would be more strongly impaired by face masks in the older than in the younger cohort. Thus, statistical evidence for age and mask effects and their interaction was estimated for one-sided hypotheses (i.e. H1_age effect_: mean _young_ > mean _old_; H1_mask effect_: mean _w/o mask_ > mean _with mask_, H1_age x mask effect_: mean _young w/o mask − with mask_ < mean _old w/o mask − with mask_). For interpersonal appraisal and attitudes towards face masks statistical evidence was estimated for two-sided hypotheses. To facilitate meta-analyses, Bayes factors in favour of the H1 (BF10) are reported along with effect sizes (*Cohen’s d,* *eta*_*p*_^*2*^, and *r*, respectively). Bayes factors and effect sizes were estimated with *JASP* (JASP Team, 2020, version 0.14.1) using one-sample and independent sample T-tests, respectively, for mask and age effects and their interaction, and repeated measures ANOVAS for emotion effects. *JASP* default priors were used for all computations. Effect sizes are reported as being small/medium/strong according to Cohen (1992) and Bayes factors are reported as anecdotal/moderate/strong/very strong/extreme evidence according to Lee and Wagenmakers (2014) as cited in Stefan et al. (2019) (except that the term”extreme” was replaced with the more commonly used term “decisive”). Raw data are reported in Addional file 2 (subjects' age and mask condition by subject, model and emotion), Additonal file 3 (selected emotion by subject, model and emotion) and Additonal file 4 (confidence ratings scaled to unity by subject, model and emotion) in the supplemental material. 

## Results

### Response times

Younger and older participants made their emotion judgements similarly quickly (mean response time over both groups and all conditions 3.4 s, Table S2 in Additional file 1 in the supplemental material) and there were no differences in response times between mask conditions (faces without masks versus faces with masks) and no age-by-mask interactions, regardless of whether absolute response times or relative increases in response times due to face masks (i.e. response time _w/o mask − with mask_ / response time _w/o mask_) were compared (all *Cohen’s d* < 0.30, all BF10 < 1 [at least anecdotal evidence against H1]). Thus, age or mask effects on emotion recognition accuracy, confidence or performance awareness cannot easily be explained by differences in response times.

### Emotion recognition accuracy, confidence and performance awareness

**Fig. 2:**
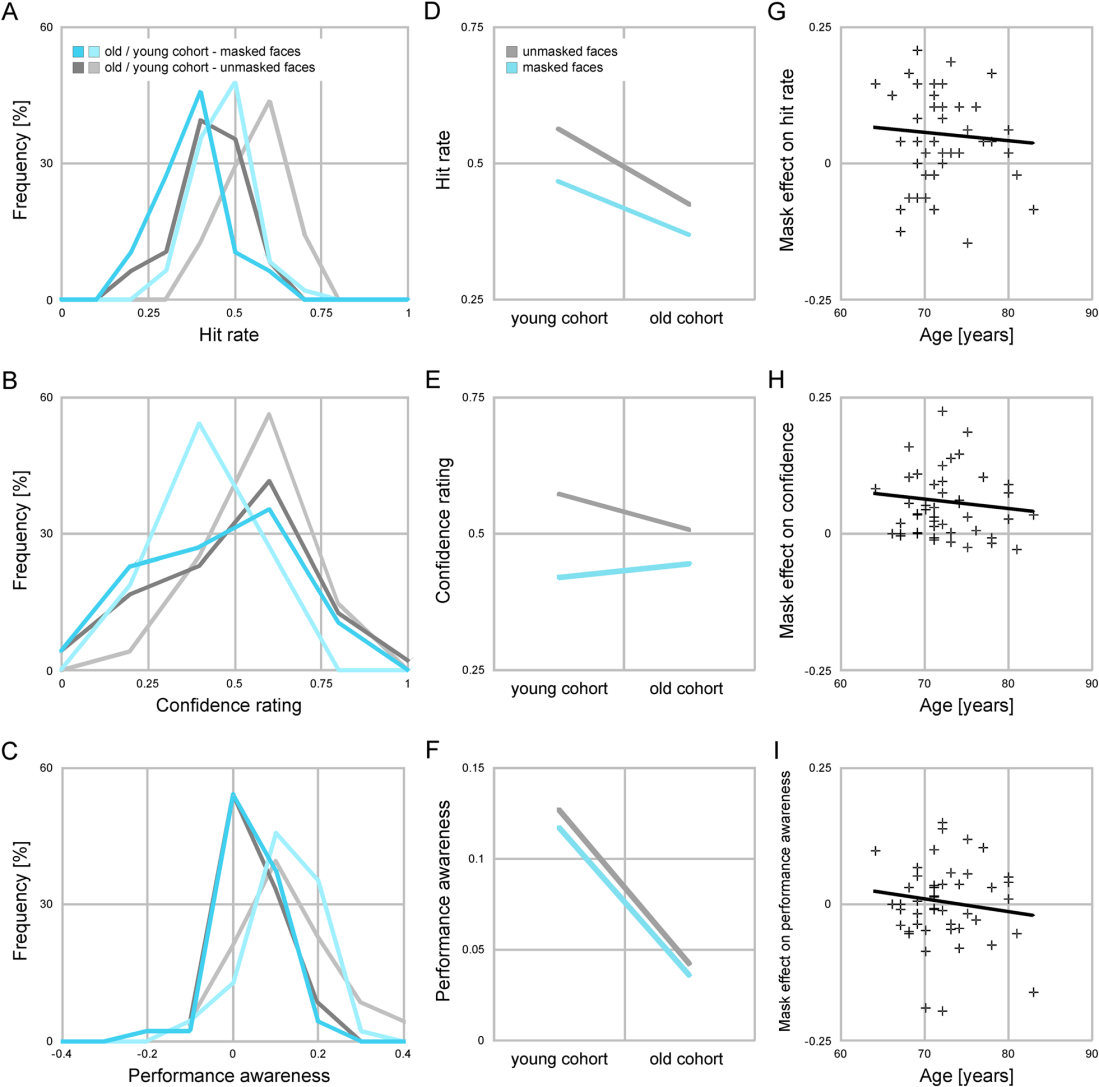
Effects of face masks on facial emotion recognition. **A**–**C**, distribution of hit rates, confidence ratings and performance awareness by mask condition and age cohort. Data are binned (bin width 0.10 for hit rates and performance awareness and 0.20 for confidence ratings). **D**–**E**, mean hit rates, confidence ratings and performance awareness by mask condition and age cohort. **G**–**I**, scatter plots showing the relation between age and mask effects on hit rates, confidence ratings and performance awareness in the old cohort. Dots represent individuals, lines are regression lines. Confidence ratings are rescaled to unity (minimal possible value 0, maximal possible value 1)

Emotion recognition accuracy was greater than chance level in both mask conditions and in both cohorts (mean hit rate over both groups and all conditions 0.46, Table 1). As predicted, emotion recognition accuracy declined with age. Young participants selected the correct emotion more often than older participants in both mask conditions (*Cohen’s d* > 1, BF10 > 100 [decisive evidence for H1] in both mask conditions, Fig. 2). Furthermore, face masks reduced emotion recognition accuracy in both cohorts (*Cohen’s d* > 0.50, BF10 > 100 [decisive evidence for H1] in both cohorts). Unexpectedly, the absolute decrease in hit rates was larger in the young than in the old cohort (0.09 versus 0.05). Thus, the data did not support the prediction that the adverse effect of face masks would be stronger in older than in younger adults (*Cohen’s d* < 0, BF10 < 0.33 [moderate evidence against H1]). To further compare the effect of face masks between cohorts we computed the *relative decline* in hit rates due to face masks, separately for each cohort (i.e. hit rate _w/o mask − with mask_ / [hit rate _w/o mask_ − chance level]). This showed that the relative decline was very similar in both cohorts (29%).

**Table 1:** Effects of face masks on emotion recognition in the young and old cohort

	Accuracy				Confidence				Performance awareness			
	Δ hit rate	*Cohen's d*	BF10	%	Rating	*Cohen's d*	BF10	%	Score	*Cohen's d*	BF10	%
*Young cohort*												
w/o mask	0.31	*3.5*	** > 100**		0.57				0.13	*1.3*	** > 100**	
With mask	0.22	*2.9*	** > 100**		0.42				0.12	*1.5*	** > 100**	
Mask effect	0.09	*1.2*	** > 100**	29%	0.15	*1.4*	** > 100**	26%	0.01	*0.09*	***0.27***	8%
*Old cohort*												
w/o mask	0.17	*1.9*	** > 100**		0.51				0.04	*0.65*	** > 100**	
With mask	0.12	*1.3*	** > 100**		0.45				0.04	*0.54*	** > 100**	
Mask effect	0.05	*0.59*	** > 100**	29%	0.06	*0.84*	** > 100**	12%	0.01	*0.07*	***0.25***	15%
*Age effect*												
w/o mask	0.14	*1.5*	** > 100**		0.06	*0.37*	1.7		0.09	*1.0*	** > 100**	
With mask	0.10	*1.2*	** > 100**		0.03	− *0.15*	***0.14***		0.08	*1.1*	** > 100**	
Mask effect	− 0.04	− *0.48*	***0.07***		− 0.03	− *0.99*	***0.04***		− 0.00	− *0.04*	***0.19***	

All comparisons are one-sided and signs indicate the direction of an effect relative to the prediction (see text). BF10 > 3 (at least moderate evidence for H1) are bold and BF10 < .33 (at least moderate evidence against H1) are bold and in italics. Δ hit, hit rate minus chance; %, relative decline due to face masks (relative to faces without masks). Confidence ratings are rescaled to unity (minimal possible value 0, maximal possible value 1)

Unlike emotion recognition accuracy the participants’ confidence that their emotion judgement in a given trial was correct did not generally decline with age (unmasked facial expressions, *Cohen’s d* > 0.20, BF10 > 1 [anecdotal evidence for H1]; masked facial expressions, *Cohen’s d* < 0, BF10 < 0.33 [moderate evidence against H1], Table 1 and Fig. 2). Face masks reduced the participants’ confidence in their judgements in both cohorts (*Cohen’s d* > 0.80, BF10 > 100 [decisive evidence for H1] in both cohorts), but again the data did not support the prediction that the adverse effect of face masks would be stronger in older than in younger adults (*Cohen’s d* < 0, BF10 < 0.10 [strong evidence against H1]).

Average confidence ratings reflect a participant’s overall confidence in their emotion judgements. They do not indicate whether a participant is aware of the *actual* correctness of their response. To obtain a measure of performance awareness (i.e. the degree to which a participant is aware of whether their response was actually correct or incorrect), we subtracted each participant’s confidence ratings in trials in which they had selected the correct response with their confidence ratings in trials in which they had selected an incorrect response. The participants’ performance awareness was greater than zero in both mask conditions and in both cohorts (all *Cohen’s d* > 0.50, BF10 > 100 [decisive evidence for H1], Table 1), but strongly declined with age (*Cohen’s d* > 0.80, BF10 > 100 [decisive evidence for H1] in both conditions, Fig. 2). Surprisingly, the data did not support the prediction that the participants’ performance awareness would be reduced by face masks, neither in the young nor in the old cohort (*Cohen’s d* < 0.20, BF < 0.33 [moderate evidence against H1] in both cohorts). As for emotion recognition accuracy and confidence, the data did not support the prediction that there would be a stronger adverse effect of face masks in older than in younger adults (*Cohen’s d* < 0, BF < 0.33 [moderate evidence against H1]).

### Correlation between emotion recognition and age in the old cohort

Between-group comparisons of accuracy, confidence and performance awareness in the younger and older cohort did not provide evidence for the prediction that the adverse effect of face masks would be larger in older than in younger adults. To test the possibility that differences in mask effects become evident only in very old individuals we examined whether the effects of face masks increased with increasing age within the old cohort (using Kendall’s tau to estimate correlation strengths to allow for nonlinear increase). This was not the case. Neither for emotion recognition accuracy, confidence or performance awareness did the adverse effect of face masks increase with age (accuracy, *Kendall’s tau* = − 0.01, BF10 = 0.15; confidence, *Kendall’s tau* = − 0.04, BF10 = 0.14; performance awareness, *Kendall’s tau* = − 0.14, BF10 = 0.08; at least moderate evidence against the H1 in all cases, Fig. 2).

### Emotion-specific effects

**Fig. 3:**
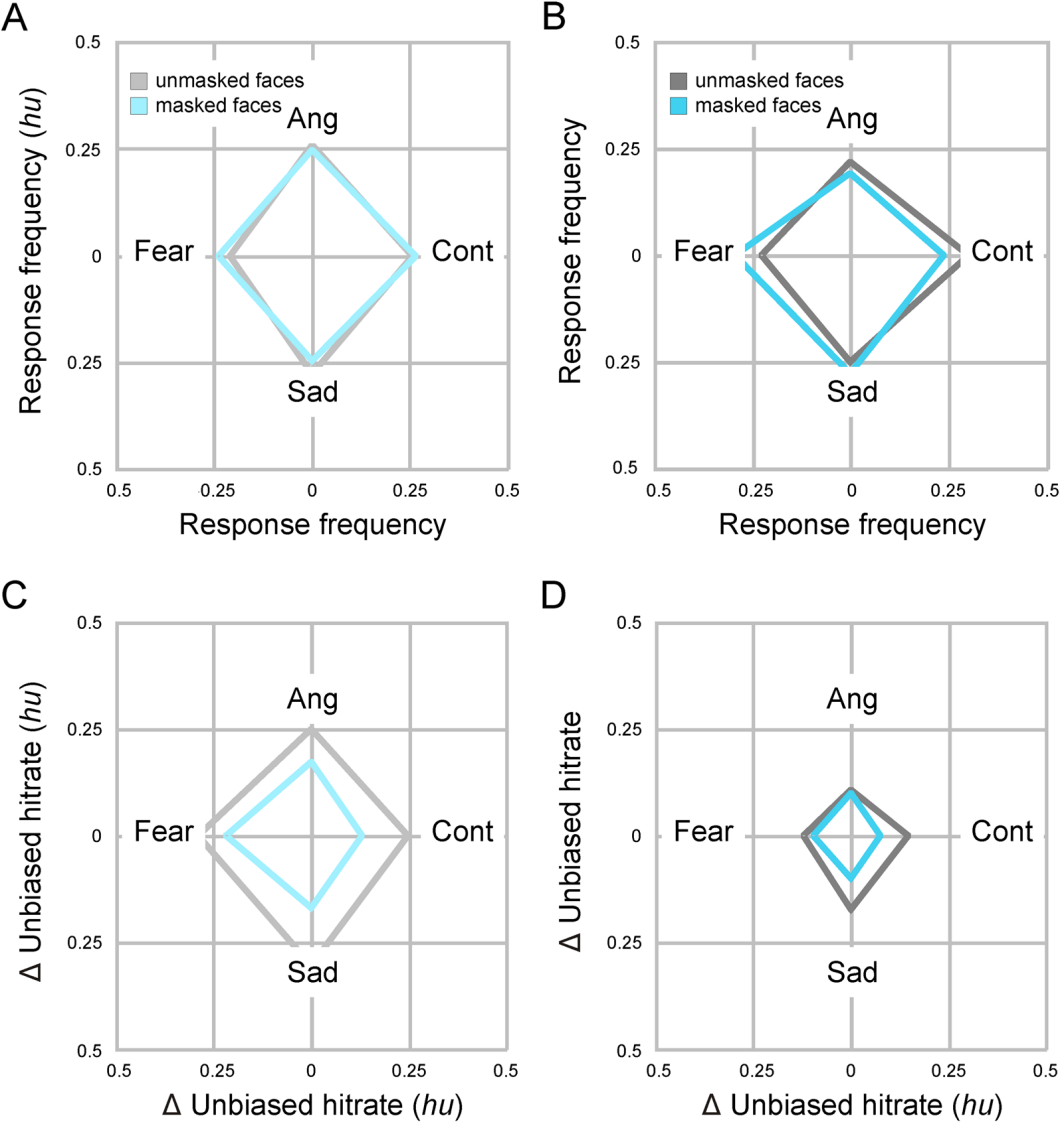
Response frequencies and unbiased hit rates for each emotion.** A, C** young cohort; **B, D** old cohort. Unbiased hit rates are subtracted with chance level

We also examined whether age and mask effects differed between emotions. First, we tested whether participants in either cohort showed a bias to select one or more response options more often than the rest. Younger participants tended to select sadness most often and fear least often in the unmasked condition (ANOVA with within-subject factor emotion; *eta*^*2*^ = 0.12, BF10 > 100 [decisive evidence for an effect], Table 2 and Fig. 3). This bias was no longer present in the masked condition (*eta*_*p*_^*2*^ = 0.02, BF < 0.10 [strong evidence for no effect]). Older participants tended to select contempt most often and anger least often in the unmasked condition (ANOVA with factor emotion; *eta*_*p*_^*2*^ = 0.10, BF10 > 100 [decisive evidence for an effect]). In the masked condition their response bias shifted from contempt to fear (*eta*_*p*_^*2*^ = 0.11, BF > 100 [decisive evidence for an effect]). Mask effects differed between emotions in the old cohort but not in the young cohort (2-way ANOVAs with within-subject factors mask and emotion; young cohort, mask-by-emotion interaction, *eta*^*2*^ = 0.06, BF = 2.7 [anecdotal evidence for an effect]; old cohort, mask-by-emotion interaction, *eta*^*2*^ = 0.13, BF > 100 [decisive evidence for an effect]), and there was a moderate age-by-mask-by-emotion interaction (3-way ANOVA with between-subject factor age and within-subject factors mask and emotion, age-by-mask-by-emotion interaction, *eta*_*p*_^*2*^ = 0.05, BF > 10 [strong evidence for an effect]), indicating that face masks modulated response biases differently in the young and old cohort.

Unbiased hit rates were greater than chance level for each and every emotion in both mask conditions and in both cohorts (all *Cohen’s d* > 0.80, BF10 > 100 [decisive evidence for H1], Table 3 and Fig. 3). Nevertheless, a strong age effect was observed for each and every emotion (all *Cohen’s d* > *0.80*, BF10 > 100 [decisive evidence for H1]). Age effects tended to be larger for *anger* and *fear* and smaller for *sadness* and *contempt* (3-way ANOVA with between-subject factor age and within-subject factors mask and emotion; age-by-emotion interaction, *eta*_*p*_^*2*^ = 0.04, BF10 = 1.6 [anecdotal evidence for an effect]). Mask effects for *anger* and fear were small in the young cohort (all *Cohen’s d* > *0.20*, BF10 > 10 [strong evidence for H1]) and absent or very small in the old cohort (all *Cohen’s d* < *0.20*, BF10 _anger_ < 0.33, BF10 _fear_ < 1 [at least anecdotal evidence against H1]). In contrast, mask effects for *sadness* and *contempt* were strong in the young cohort (all *Cohen’s d* > *0.80*, BF10 > 100 [decisive evidence for H1]), and medium strong in the old cohort (all *Cohen’s d* > *0.50*, BF10 _sadness_ > 30, BF _contempt_ > 100 [at least very strong evidence for H1]). Mask effects differed between emotions (3-way ANOVA with between-subject factor age and within-subject factors mask and emotion; mask-by-emotion interaction, eta_*p*_^2^ = 0.05, BF10 = 10 [strong evidence for an effect]) but the data did not provide evidence for an age-by-mask-by-emotion effect (eta_*p*_^2^ < 0.001, BF < 0.10 [strong evidence for no effect]).

**Table 2:** Effects of face masks on response frequencies in the young and old cohort

	Response frequency						
	Anger	Fear	Sadness	Contempt	*F*	*eta*_*p*_^*2*^	*BF10*
*Young cohort*							
w/o mask	0.26	0.21	0.28	0.26	*6.4*	*0.12*	** > 100**
With mask	0.25	0.24	0.25	0.27	< *1*	*0.02*	***0.09***
Mask effect	− 0.01	0.03	− 0.03	0.01	*2.9*	*0.06*	2.7
*Old cohort*							
w/o mask	0.22	0.23	0.25	0.30	*5.2*	*0.10*	** > 100**
With mask	0.19	0.30	0.28	0.24	*5.7*	*0.11*	** > 100**
Mask effect	− 0.03	0.07	0.03	− 0.06	*7.1*	*0.13*	** > 100**
*Age effect*							
Mask effect	− 0.02	0.04	0.06	− 0.07	4.8	0.05	**12**

Positive signs indicate higher response frequencies for masked facial expressions / larger mask effects in the old cohort. BF10 > 3.0 (at least moderate evidence for an effect) are bold and BF10 < 0.30 (at least moderate evidence for no effect) are bold and in italics

**Table 3:** Emotion-specific effects of face masks on emotion recognition accuracy in the young and old cohort

	Accuracy															
	Anger				Fear				Sadness				Contempt			
	*Δ hu*	*Cohen's d*	BF10	%	*Δ hu*	*Cohen's d*	BF10	%	*Δ hu*	*Cohen's d*	BF10	%	*Δ hu*	*Cohen's d*	BF10	%
*Young cohort*																
w/o mask	0.25	*1.7*	**> 100**		0.29	*2.0*	**> 100**		0.30	*2.4*	**> 100**		0.25	*1.9*	**> 100**	
With mask	0.17	*1.7*	**> 100**		0.22	*1.7*	**> 100**		0.17	*1.7*	**> 100**		0.13	*1.3*	**> 100**	
Mask effect	0.08	*0.45*	**22**	31%	0.07	*0.43*	**14**	25%	0.14	*0.86*	**> 100**	45%	0.12	*0.85*	**> 100**	48%
*Old cohort*																
w/o mask	0.11	*1.1*	**> 100**		0.12	*1.0*	**> 100**		0.17	*1.3*	**> 100**		0.15	*1.4*	**> 100**	
With mask	0.10	*0.96*	**> 100**		0.10	*0.96*	**> 100**		0.10	*1.0*	**> 100**		0.07	*0.82*	**> 100**	
Mask effect	0.01	*0.07*	***0.25***	8%	0.02	*0.17*	0.55	20%	0.08	*0.51*	**53**	44%	0.07	*0.57*	**> 100**	50%
*Age effect*																
Mean	0.11	*1.2*	**> 100**		0.15	*1.5*	**> 100**		0.10	*1.2*	**> 100**		0.08	*0.92*	**> 100**	
Mask effect	0.07	− *0.46*	***0.07***		0.05	− *0.31*	***0.09***		0.06	− *0.39*	***0.08***		0.05	− *0.34*	***0.09***	

All comparisons are one-sided and signs indicate the direction of an effect relative to the prediction (see text). BF10 > 3 (at least moderate evidence for H1) are bold and BF10 < .33 (at least moderate evidence against H1) are bold and in italics. Δ *hu*, unbiased hit rate minus chance; %, relative decline due to face masks (relative to faces without masks)

**Fig. 4:**
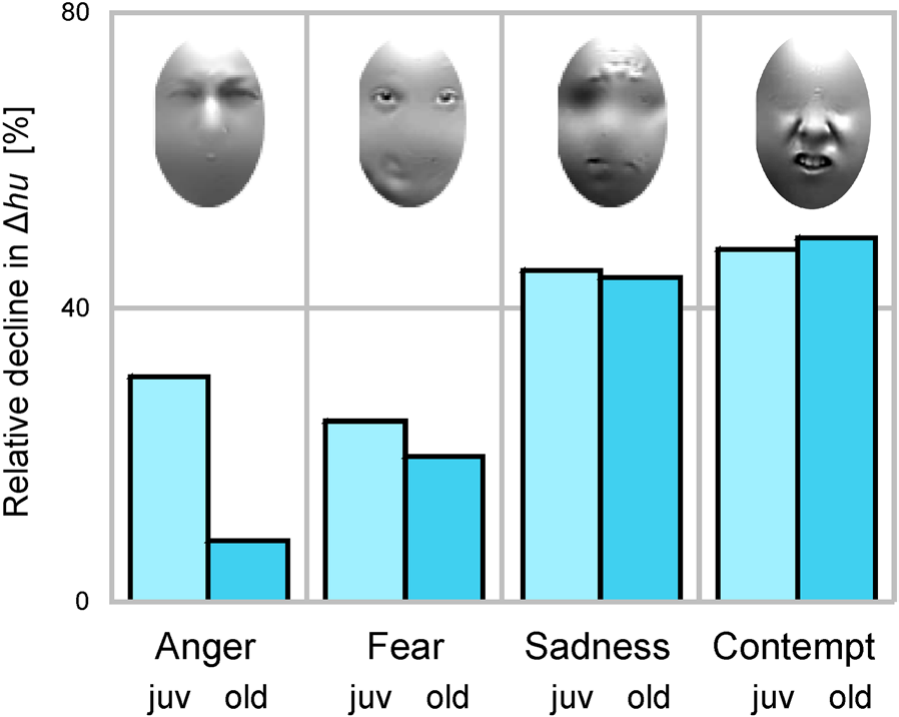
Relative decline due to face masks in unbiased hit rates for each emotion. Inserts show the facial features most often used by participants to decode each emotion , adapted with permisssion from Smith et al. 2005 (the right most insert shows features used to decode disgust). juv, young cohort; old, old cohort

To further compare emotion-specific mask effects between cohorts we computed the relative decline in emotion recognition accuracy due to face masks, separately for each emotion and cohort. This revealed that relative declines for *anger* and *fear* were smaller in the older than in the younger cohort, while relative declines for *sadness* and *contempt* were equally large in both cohorts (for *contempt* the relative decline was even marginally larger in the older than in the younger cohort, Table 3). When emotions were ordered by relative decline (i.e. *anger*-*fear*-*sadness*-*contempt*), the average emotion-to-emotion increase in decline was 6% in the young cohort and 14% in the old cohort (Fig. 4).

### Interpersonal appraisal

The participants’ initial appraisal of the models did not differ between age cohorts and was unaffected by face masks (all *Cohen’s d* |d|< 0.50, BF10 < 1, two-sided [at least anecdotal evidence against H1], Table S3 in Additional file 1 in the supplemental material) with one exception: Older participants felt closer to models who were wearing a face mask than younger participants (*Cohen’s d* |*d*|= 0.54, BF10 > 3, two-sided [moderate evidence for H1]). Evidence concerning age-by-mask interactions was inconclusive in all cases (Table S3 in Additional file 1 in the supplemental material).

### Attitudes towards face masks

Younger and older participants did not differ with respect to how much they felt that face masks impair their understanding of other people’s emotions (*atom factor 1*, *perceived impairment*, score _young_ = 25.9, score _old_ = 26.4 [maximal possible score = 42], *Cohen’s |d*| = 0.07, BF10 = 0.28, two-sided [moderate evidence against H1]) or how useful they regarded face masks in confining the COVID-19 pandemic (*atom factor 2*, *perceived utility,* score _young_ = 25.6, score _old_ = 26.7 *Cohen’s |d|* = 0.15, BF10 = 0.41, two-sided [anecdotal evidence against H1]).

### Individual differences

Finally, we tested whether there were interindividual differences in mask effects on emotion recognition within each age cohort that could be explained by the participants’ attitudes towards face masks. For this, we fitted a linear model with factors *perceived impairment* (*atom* factor 1) and *perceived utility* (*atom* factor 2) to each measure (hit rate _w/o mask − with mask,_ confidence _w/o mask − with mask_, performance awareness _w/o mask − with mask_), separately for each cohort. Evidence for partial correlations between one or both *atom* factors and emotion recognition measures was inconclusive in most cases (0.33 < BF < 1, Table 4). Three relations were supported by anecdotal evidence: (i) a negative relation between *perceived impairment* and mask effects on performance awareness in the young cohort, (ii) a positive relation between *perceived utility* and mask effects on performance awareness in the young cohort and (iii) a positive correlation between *perceived impairment* and mask effects on perceived closeness in the old cohort (BF > 1 in all cases). Additionally, one relation was supported by moderate evidence: a positive correlation between *perceived impairment* and mask effects on the participants’ confidence in their emotion judgements in the old cohort (BF = 4.8).

**Table 4:** Relation between attitudes towards face masks as assessed with the *atom* questionnaire and face mask effects on emotion recognition and interpersonal appraisal

	Accuracy		Confidence		Awareness		Trustability		Liking		Closeness	
	*r*	BF10	*r*	BF10	*r*	BF10	*r*	BF10	*r*	BF10	*r*	BF10
*Young cohort*												
Impairment	*0.29*	0.79	*0.16*	0.40	*− 0.21*	1.3	*− 0.08*	0.34	*− 0.12*	0.43	*− 0.04*	***0.24***
Utility	*0.14*	0.42	*0.26*	0.60	*0.26*	1.8	*0.16*	0.42	*0.15*	0.48	*0.02*	***0.24***
*Old cohort*												
Impairment	*− 0.13*	***0.30***	*0.37*	**4.8**	*− 0.06*	***0.24***	*0.21*	0.83	*0.25*	0.96	*0.31*	1.9
Utility	*− 0.07*	***0.26***	*− 0.05*	0.59	*− 0.08*	***0.25***	*− 0.15*	0.62	*− 0.08*	0.46	*− 0.06*	0.52

BF10 > 3 (at least moderate evidence for a relation) are bold and BF10 < 0.33 (at least moderate evidence for no relation) are bold and in italics. *r*, partial correlation coefficient

Thus, in the young cohort face masks reduced the performance awareness of participants who regard face masks as highly useful, possibly indicating that these participants “ignored” their incorrect responses in the masked condition (recall that across all younger participants performance awareness was not reduced by face masks). On the contrary, face masks increased the performance awareness of participants who reported to feel more impaired in understanding others by face masks, possibly indicating that these participants were more sensitive to their incorrect responses in the masked condition. In the old cohort, participants who reported feeling more impaired in understanding others were less confident when judging facial expressions covered by face masks and felt less close to models wearing a face mask than to models not wearing a face mask. However, this did not reflect their actual emotion recognition performance (accuracy or performance awareness).

## Discussion

The aim of the current study was to examine and compare the effects of surgical face masks on facial emotion recognition in younger and older adults. Surgical face masks cover up to 70% of the face (Carbon, 2020) and render almost all visual information below the eye region inaccessible. Studies that have mapped the informational content of affective facial expressions suggest that the lower part of the face carries more diagnostic information than the upper part across a wide range of emotions (Blais et al., 2012; Kotsia et al., 2008; Wegrzyn et al., 2017). Thus, surgical face masks can be expected to have a profound effect on facial emotion recognition. Not surprisingly, six of the seven published studies that have examined the effect of surgical face masks on facial emotion accuracy (by October 2021) conclude that face masks significantly reduce emotion recognition accuracy (Bani et al., 2021; Carbon, 2020; Carbon & Serrano, 2021; Gori et al., 2021; Grundmann et al., 2021; Noyes et al., 2021).

Three of these studies report mean hit rates (or number of errors) for both fully visible and masked facial expressions. In two of these studies, face masks reduced mean hit rates from roughly 0.80 for fully visible facial expressions to roughly 0.70 for masked facial expressions (Bani et al., 2021; Gori et al., 2021). In the third study, face masks reduced the mean hit rate from roughly 0.70 to roughly 0.50 (Grundmann et al., 2021). In the current study, hit rate decreased from 0.49 for fully visible facial expressions to 0.42 for masked facial expressions. Thus, the absolute decline in hit rates due to face masks was smaller in the current study. However, baseline performance (hit rates for fully visible facial expressions) and chance level also varied between studies, making it difficult to compare absolute hit rates. The relative decline due to face masks (hit rate _w/o mask − with mask_ / [hit rate _w/o mask_ − chance level]) ranged from roughly 20% in the study by Gori et al. (2021) (chance level = 0.20) over roughly 22% in the study by Bani et al. (2021) (chance level = 0.25) and 29% in the current study (chance level = 0.25) to roughly 33% in the study by Grundmann et al. (2021) (chance level = 0.11). Thus, the relative decline in hit rates due to face masks appears quite similar across studies.

Information-mapping studies that compared different facial expressions suggest that facial expressions of anger and fear carry less diagnostic information in the mouth region than facial expressions of disgust (Smith et al., 2005; Wegrzyn et al., 2017). In line with this, studies that examined how face masks affect the detection of specific emotions report that face masks impair the detection of anger (Noyes et al., 2021) and fear (Bani et al., 2021; Carbon, 2020) less than that of other emotions. In the two studies that included facial expressions of disgust (Carbon, 2020; Noyes et al., 2021) this was the emotion whose detection was most severely impaired by face masks. Interestingly, the detection of disgust was not impaired by sunglasses (Noyes et al., 2021). This pattern was replicated by the current study: the detection of anger and fear was less affected by face masks than the detection of contempt.

The current study particularly addressed the question whether surgical face masks reduce emotion recognition accuracies in younger and older adults to different degrees. In line with previous studies (Ruffman et al., 2008; Goncalves et al., 2018; Hayes et al., 2020) we found that emotion recognition accuracy considerably declined with age. Interestingly, previous studies also suggest that older adults do not only show a general decline in emotion recognition accuracy but that they also differ from younger adults in the way they use affective information in facial expressions. Older adults spend more time scanning the mouth region of fully visible faces than younger adults (Grainger & Henry, 2020). At the same time, they seem to be relatively more impaired in decoding anger and fear (emotions that are mainly encoded in the eye region, see above) than in decoding disgust (an emotion that is mainly encoded in the mouth region, see above) (Ruffman et al. 2008; Goncalves et al. 2018; Hayes et al. 2020). This suggests that older people rely more on affective information in the mouth region than younger people. Thus, we predicted that occluding the mouth region by a face mask should impact emotion recognition accuracy disproportionally more strongly in older adults.

This was not the case. Contrary to our prediction the absolute decline in emotion recognition accuracy due to face masks was weaker in older than in younger adults (0.05 versus 0.09). Moreover, a weaker absolute decline in older than in younger adults was observed for each and every emotion (*anger*, 0.01 versus 0.08; *fear*, 0.02 versus 0.07; *sadness*, 0.08 versus 0.14; *contempt*, 0.07 versus 0.12). This suggests that, in absolute terms, older adults use information from the mouth region less efficiently than younger adults. However, considering that overall emotion recognition accuracy declines with age, it is perhaps not surprising that information from the mouth region was, in absolute terms, less efficiently used by older adults. Looking at the relative decline in each age cohort suggests a slightly different picture. First, the relative decline across all emotions was very similar in both cohorts (29%). Second, in both cohorts, the relative decline was small for facial expressions of anger and fear (less than 33%) and larger for facial expressions of sadness and contempt (up to 50%). Third, for facial expressions of anger and fear, the relative decline was considerably smaller in older than in younger participants, while for facial expressions of sadness and contempt the relative decline was very similar between cohorts (for contempt the relative decline was even marginally larger in older than in younger participants) (see Fig. 3). This suggests that for emotions that are mainly encoded in the mouth region (contempt and, to some extent, sadness) the relative contribution of the mouth region was similar in older and younger participants (50% / 48% for contempt and 44% / 45% for sadness in the old/young cohort). Surprisingly, for emotions that are mainly encoded in the eye region (fear and anger) the relative contribution of the mouth region was *less* in older than in younger participants (20% / 25% for fear and 8% / 31% for anger in the old /young cohort). In other words, if there is relatively little information in the mouth region (as in facial expressions of fear and anger) older people seem to neglect this information.

Interestingly, a pattern similar to that observed in the current study was evident in a study that examined the effect of face masks on emotion recognition in school children (Carbon & Serrano, 2021). In that study, face masks profoundly reduced the detection of disgust (by almost 90%) but even slightly improved the detection of anger (by almost 5%), with intermediate effects for fear (reduction by roughly 13%) and sadness (reduction by roughly 15%) (chance level = 0.17). This anger-fear-sadness-disgust gradient appears to be even steeper (average increase in relative decline from emotion to emotion 25%) than the anger-fear-sadness-contempt gradient observed for old adults in the current study (average increase in relative decline from emotion to emotion 15%). For comparison, in a previous study by the same first author that used a very similar protocol in adult participants (Carbon, 2020) the anger-fear-sadness-disgust gradient was about 15%. It must be noted though that this interpretation is tentative and based on uncorrected hit rates (reported in Fig. 2 in Carbon & Serrano, 2021, and Carbon, 2020, respectively) which might be confounded by response biases.

Remarkably, age effects were larger than mask effects in the current study (difference in absolute hit rates between age cohorts 0.12; average decrease in absolute hit rates due to face masks, 0.07). This is different to results reported by Grundmann et al. (2021). In their study, absolute hit rates differed between younger and older participant by 0.13 (0.65 versus 0.52) and face masks led to a reduction of 0.20 (from 0.70 to 0.50). This corresponds to a relative reduction of roughly 25% due to age and roughly 33% due to face masks (chance level = 0.11). This difference between the two studies might be due to differences in the stimulus sets. Unlike the stimulus set used in the current study, the stimulus set used by Grundmann et al. (2021) included facial expressions of surprise and happiness, two emotions that are primarily (and more than any of the four emotions examined in the current study) encoded in the mouth region (Smith et al., 2005). In the current study, age effects were smaller and mask effects were larger for facial expressions that were mainly encoded in the mouth region (sadness and contempt). Thus, age effects might be even smaller, and mask effects might be even larger, for facial expressions of happiness and surprise. This could lead to overall smaller age and larger mask effects as observed by Grundmann et al. (2021).

Grundmann et al. (2021) also observed a moderate age-by-mask interaction, with a stronger decline due to face masks in older than in younger participants. Again, this might be explained by differences between the stimulus sets. Extrapolating the anger-fear-sadness-contempt gradient observed in the current study would predict that for emotions that are primarily encoded in the mouth region such as surprise and happiness the decline in emotion recognition accuracy due to face masks might be larger in older than in younger adults. Unfortunately, Grundmann et al. (2021) do not report emotion-specific effects.

The second unpredicted finding of the current study concerns the participants’ performance awareness (i.e. the difference between a participant’s confidence ratings in trials in which they had selected the correct response and their confidence ratings in trials in which they had selected an incorrect response). In contrast to the participants’ emotion recognition accuracy and confidence their performance awareness did not decline when the amount of available facial information was reduced by a face mask. In social contexts, performance awareness (knowing if one does or does not understand another person’s signals) permits individuals to navigate smoothly in their social environment and to build functional social networks (Anders et al., 2016). At the neural level, performance awareness seems to be mediated by neurons in the ventral striatum that signal whether sufficient information is available to accurately decode a signal (Anders et al., 2016; Daniel & Pollmann, 2012; Hebart et al., 2016). The current study suggests that the reliability of this system decreases with age. Unexpectedly, however, the system seems to be robust against signal degradation and to work well even when the available information is reduced by more than 50%.

Finally, two minor findings should be mentioned. Both the participants’ initial appraisal of the models and their attitudes towards face masks as measured with the *atom* (*perceived impairment by face masks* and *perceived utility of face masks*) did not differ between age cohorts, and the participant’s initial appraisal of the models was unaffected by face masks. Nevertheless, older participants who felt more impaired in understanding others’ emotions by face masks were less confident when judging a masked model’s emotion and felt less close to models wearing a face mask than to models whose face was fully visible. This suggests that in some older participants the perceived costs of face masks are higher than in others. However, in these adults perceived costs did not seem to reflect actual impairments in understanding other people’s emotions.

### Limitations and future directions

The current study examined the impact of face masks on emotion recognition in dynamic facial expressions of lay models. Importantly, models were explicitly instructed not to pose facial expressions but to express their genuine affective feelings. Thus, the ecological validity of the current study might be higher than that of previous studies, and baseline performance in the current study (average hit rate 0.56 in the younger cohort) might approximate real life performance more closely than that of previous studies (0.70 and higher, see above). Ecological validity of studies examining face mask effects is particularly important as both the study by Grundmann et al. (2021) and the current study indicate that, in absolute terms, older adults are considerably worse than younger adults in discriminating masked facial expressions. If experimentally derived estimates of emotion recognition accuracy substantially overestimate real life performance, vulnerable groups who show hit rates well above chance in experimental settings might actually perform at chance level in real life.

However, the potentially high ecological validity of the current study also entailed a major limitation. Not all participants achieved hit rates that were larger than chance level for each and every emotion, particularly in the old cohort. As a consequence, relative declines in hit rates due to face masks could only be computed at group level, preventing statistical analyses. Future studies might thus aim to select stimuli such that hit rates are above chance level in all conditions and for each individual participant.

A second, related, limitation is that the current study did not include facial expressions of positive affect. The LUV does not include facial expressions of positive affect because facial expressions of happiness can often be perfectly discriminated from facial expressions of negative affect, leading to very different hit rates for different emotions. Future studies, particularly if they examine effects of masking, should include a well balanced set of positive emotions, preferably controlling for the amount of diagnostic information encoded in the eye and mouth region across emotional valences.

Third, face masks in the current study (as in all previous studies we are aware of) were digitally added to pre-recorded videos. Thus, we actually examined the decoding of facial signals that were artificially degraded. It seems very likely that in face-to-face communication senders adapt their expressive behaviour when wearing a face mask. Thus, studies published so far might overestimate the effect of face masks on facial communication.

Finally, it should be noted that in the current study more than 80 percent of the participants in the old cohort were retired while participants in the young cohort were either employed or students. While it is currently unknown whether and when retirement fosters or lowers social skills it would be highly interesting to see whether the age-related effects observed in the current study are more likely due to environmental or neurophysiological causes.

## Conclusion

In sum, the current study has two important implications. First, it confirms that occluding part of the face with a surgical face mask impairs the understanding of facial signals of affect. Contrary to our prediction, this effect was not larger in older than in younger adults, suggesting that older adults might actually rely *less* on information in the mouth region than younger adults, at least for some facial expressions. Nevertheless older adults performed worse than younger adults when confronted with masked facial expressions. Second, it suggests that older adults are less aware than younger adults of whether their understanding of another person’s affect is correct or not, but that performance awareness is unaffected by face masks, even in very old adults. We believe that the latter finding is particularly encouraging in the context of the current pandemic: Face masks might impair communication, but individuals are still aware of whether the flow of information between them is sufficient to permit mutual understanding, allowing them to adapt their behaviour when it is not.

## Supplementary Information

**Additional file 1:** Table S1-S3.

**Additional file 2:** Mask condition by subject, model and emotion.

**Additional file 3:** Subjects' emotion judgements by subject, model and emotion.

**Additional file 4:** Subjects' confidence ratings (scaled to unity) by subject, model and emotion.

## Data Availability

All data analysed during this study are included in this published article and its supplementary information files.
