# Supplementary material for: Surgical face masks do not impair the decoding of facial expressions of negative affect more severely in older than in younger adults
Source: Cogn Res Princ Implic. 2022 Jul 16;7:63. doi: 10.1186/s41235-022-00403-8 (PMC9287709; doi:10.1186/s41235-022-00403-8)
Supplement: Supplementary file 1 — Additional file 1: Table S1-S3. [file 41235_2022_403_MOESM1_ESM.doc]

**Supplemental material** *Henke et al.*

| **Item** |  | **Loading** | |
| --- | --- | --- | --- |
|  |  | **F1** | **F2** |
| 1 | Ich finde, Masken beeinträchtigen das menschliche Miteinander. | **.60** | .04 |
| 2 | Ich glaube, man kann auch mit Masken gute Begegnungen mit anderen Menschen haben. | .27 | .47 |
| 3 | Ich finde es befremdlich, wenn ich mit einem fremden Menschen spreche, dessen Gesicht ich nicht vollständig sehen kann, weil es von einer Maske bedeckt ist. | **.71** | .07 |
| 4 | Menschen mit Masken wirken auf mich manchmal gefühllos. | **.62** | .10 |
| 5 | Ich sehne mich nach Begegnungen mit Menschen, deren Gesicht nicht von einer Maske bedeckt ist. | **.48** | .04 |
| 6 | Ich habe Schwierigkeiten, die Gefühle von Menschen zu erkennen, die eine Maske tragen. | **.54** | .20 |
| 7 | Ich denke, es gibt zur Bekämpfung der Pandemie bessere Maßnahmen als das Tragen einer Maske. | -.27 | .35 |
| 8 | Um mich und andere zu schützen, trage ich manchmal auch dann eine Maske, wenn es nicht vorgeschrieben ist. | .19 | **.56** |
| 9 | Ich glaube, dass Masken in Gesprächssituationen sicher vor Ansteckungen schützen können. | .05 | **.81** |
| 10 | Ich fühle mich geschützt, wenn jemand, mit dem ich spreche, eine Maske trägt. | .10 | **.72** |
| 11 | Wenn ich jemanden sehe, dessen Maske verrutscht ist, halte ich Abstand. | .03 | **.61** |
| 12 | Ich denke, im Freien ist es unnötig, eine Maske zu tragen. | -.39 | .23 |
| **Table S1. Loadings of each item of the *atom* questionnaire on the two factors**. Exploratory factor analysis favoured a two-factorial solution (*Kaiser-Guttman criterion*, *Horn‘s parallel analysis*). The table shows loadings for the first two factors after promax rotation. Cronbach’s α = .71 for items 1-6 (factor 1, *perceived impairment by face masks*) and *Cronbach’*s α = .72 for items 7-12 (factor 2, *perceived utility of face masks*). Factor loadings of λ > .48 are bold. F1, factor 1, F2, factor 2. | | | |

|  | **Young cohort** | | | | | | **Old cohort** | | | | | | **Age effect** | | | | | |
| --- | --- | --- | --- | --- | --- | --- | --- | --- | --- | --- | --- | --- | --- | --- | --- | --- | --- | --- |
|  | rt | *d* | BF10 | % | *d* | BF10 | rt | *d* | BF10 | % | *d* | BF10 | rt | *d* | BF10 | % | *d* | BF10 |
| w/o mask | 2.7 s |  |  |  |  |  | 3.3 s |  |  |  |  |  | 0.6 s | *.28* | .87 |  |  |  |
| with mask | 3.0 s |  |  |  |  |  | 4.8 s |  |  |  |  |  | 1.8 s | *.26* | .78 |  |  |  |
| mask effect | 0.3 s | *.10* | .30 | 27% | *.23* | .94 | 1.5 s | *.18* | .56 | 57% | *.18* | .56 | 1.2 s | *.18* | .48 | 30% | *.12* | .36 |
| **Table S2. Response times.** Comparisons are one-sided and signs indicate the direction of an effect relative to the prediction (i.e., rt _young_ < rt _old_; rt _w/o mask_ < rt _with mask_; rt _young with mask – w/o mask_ < rt _old with mask – w/o mask_). Relative increases due to face masks are scaled to response times for faces without masks. BF10 > 3 (at least moderate evidence for H1) are bold and BF10 < .33 (at least moderate evidence against H1) are underlined. rt, mean response time; *d*, *Cohen’s d;* % mean relative increase due to face masks. | | | | | | | | | | | | | | | | | | |

|  | **Trustability** | | | | **Likability** | | | | **Closeness** | | | |
| --- | --- | --- | --- | --- | --- | --- | --- | --- | --- | --- | --- | --- |
|  | rating | *d* | BF10 | % | rating | *d* | BF10 | % | rating | *d* | BF10 | % |
| **Young cohort** |  |  |  |  |  |  |  |  |  |  |  |  |
| w/o mask | .14 |  |  |  | .07 |  |  |  | -.28 |  |  |  |
| with mask | .11 |  |  |  | .05 |  |  |  | -.32 |  |  |  |
| mask effect | .03 | *.12* | .22 | 21% | .02 | *.09* | .19 | 29% | .05 | *.18* | .32 | 18% |
| **Old cohort** |  |  |  |  |  |  |  |  |  |  |  |  |
| w/o mask | .07 |  |  |  | .04 |  |  |  | -.19 |  |  |  |
| with mask | .11 |  |  |  | .11 |  |  |  | -.13 |  |  |  |
| mask effect | -.04 | *-.16* | .28 | 57% | -.07 | *-.29* | .97 | >100 | -.06 | *-.26* | .68 | 32% |
| **Age effect** |  |  |  |  |  |  |  |  |  |  |  |  |
| w/o mask | .03 | *.25* | .42 |  | .03 | *.09* | .23 |  | -.09 | *-.25* | .41 |  |
| with mask | 0 | *.02* | .22 |  | -.06 | *-.21* | .35 |  | -.19 | *-.54* | **4.7** |  |
| age x mask effect | -.07 | *-.29* | .51 |  | -.09 | *-.37* | .92 |  | -.11 | *-.44* | 1.6 |  |
| **Table S3. Effects of face masks on interpersonal appraisal in the young and old cohort.** Comparisons are two-sided (i.e., H1: effect ≠ 0, H0: effect = 0). Positive signs indicate more positive ratings for unmasked than for masked faces and more positive ratings in the young than in the old cohort. Percentages are relative to the mean for unmasked faces in each cohort. BF10 > 3 (at least moderate evidence for H1) are bold and BF10 < .33 (at least moderate evidence against H1) are underlined. Ratings scales are rescaled to range from -1 to 1. *d*, *Cohen’s d.* | | | | | | | | | | | | |
